# Insulin Resistance at the Crossroad of Alzheimer Disease Pathology: A Review

**DOI:** 10.3389/fendo.2020.560375

**Published:** 2020-11-05

**Authors:** Jorge Berlanga-Acosta, Gerardo Guillén-Nieto, Nadia Rodríguez-Rodríguez, Maria Luisa Bringas-Vega, Diana García-del-Barco-Herrera, Jorge O. Berlanga-Saez, Ariana García-Ojalvo, Mitchell Joseph Valdés-Sosa, Pedro A. Valdés-Sosa

**Affiliations:** ^1^The Clinical Hospital of Chengdu Brain Science Institute, MOE Key Lab for Neuroinformation, University of Electronic Science and Technology of China, Chengdu, China; ^2^Tissue Repair and Cytoprotection Research Group, Center for Genetic Engineering and Biotechnology, Havana, Cuba; ^3^Cuban Neurosciences Center, Cubanacan, Havana, Cuba; ^4^Applied Mathematics Department, Institute of Mathematics, Federal University of Rio de Janeiro, Rio de Janeiro, Brazil

**Keywords:** Alzheimer's disease, neurodegeneration, β-amyloid plaques, neurofibrillary tangles, central insuline resistance

## Abstract

Insulin plays a major neuroprotective and trophic function for cerebral cell population, thus countering apoptosis, beta-amyloid toxicity, and oxidative stress; favoring neuronal survival; and enhancing memory and learning processes. Insulin resistance and impaired cerebral glucose metabolism are invariantly reported in Alzheimer's disease (AD) and other neurodegenerative processes. AD is a fatal neurodegenerative disorder in which progressive glucose hypometabolism parallels to cognitive impairment. Although AD may appear and progress in virtue of multifactorial nosogenic ingredients, multiple interperpetuative and interconnected vicious circles appear to drive disease pathophysiology. The disease is primarily a metabolic/energetic disorder in which amyloid accumulation may appear as a by-product of more proximal events, especially in the late-onset form. As a bridge between AD and type 2 diabetes, activation of c-Jun N-terminal kinase (JNK) pathway with the ensued serine phosphorylation of the insulin response substrate (IRS)-1/2 may be at the crossroads of insulin resistance and its subsequent dysmetabolic consequences. Central insulin axis bankruptcy translates in neuronal vulnerability and demise. As a link in the chain of pathogenic vicious circles, mitochondrial dysfunction, oxidative stress, and peripheral/central immune-inflammation are increasingly advocated as major pathology drivers. Pharmacological interventions addressed to preserve insulin axis physiology, mitochondrial biogenesis-integral functionality, and mitophagy of diseased organelles may attenuate the adjacent spillover of free radicals that further perpetuate mitochondrial damages and catalyze inflammation. Central and/or peripheral inflammation may account for a local flood of proinflammatory cytokines that along with astrogliosis amplify insulin resistance, mitochondrial dysfunction, and oxidative stress. All these elements are endogenous stressor, pro-senescent factors that contribute to JNK activation. Taken together, these evidences incite to identify novel multi-mechanistic approaches to succeed in ameliorating this pandemic affliction.

## Introduction

Brain metabolism accounts for 50% of total body glucose as its main energy source having neurons with the highest energy demand (1–3). Neuronal energetic dyshomeostasis is behind the onset of numerous devastating pathologic nervous conditions, characterized by reduced neuronal survival and an overall deterioration of cognitive abilities (4).

Insulin physiology is as broad as meaningful. It is a sort of central axis transecting nutrients metabolic homeostasis to systemic growth and development, in both embryonic and extraembryonic life. The insulin physiological umbrella also regulates fertility and life span. Alike, this system turns critical for neuronal growth, connectivity, survival, and integral brain function. This definition emphasizes the catastrophic outcomes that may result from peripheral and/or central insulin resistance (IR) (5). In consonance with this, the postulate that IR/energy metabolic derangements are pathogenically engaged as a causative factor for Alzheimer's disease (AD) and other forms of dementia stands as an exciting puzzle in which an upstream metabolic disorder may probably act as the proximal trigger in a web of interconnected pathogenic circuits and vicious circles (6).

AD is a devastating neurodegenerative condition caused by an intricate and multifaceted pathophysiology, standing as the most common cause of degenerative dementia (7). A collection of histopathological, imaging, metabolic, and molecular markers characterize AD, including cerebral cortical and subcortical atrophy, neuronal loss, synaptic terminal damages, microvascular damages, different inflammatory reactions including reactive astrocytosis, and significantly hyperphosphorylated Tau and increased amyloid beta (Aβ) deposits (8).

Aside from the epidemiological association, *in vitro* and *in vivo* studies have offered significant insight into the impact of energy dyshomeostasis on the onset and progression of AD pathology. Although the biological interdependence of central IR and AD is not completely elucidated, impaired insulin signaling associated with other neuronal metabolic derangements, a local inflammatory environment with the associated oxidative stress, appear to be crucial drivers to disrupt neuronal energy homeostasis (9).

From the clinical point of view, AD has major presentation forms. The less frequent familial or early-onset AD form (FAD) debuts in subjects younger than 65 years and is conditioned by inherited mutations in three main genes: amyloid precursor protein (APP), presenilin-1 (PSEN1), and presenilin-2. Alternatively, the sporadic (SAD) or late-onset AD (LOAD) includes most of the AD subjects' universe, mostly diagnosed after the sixth decade of life. Apolipoprotein E (APOE 19q32.13) epsilon4 allele has been identified as the main genetic risk factor for the sporadic form. Of note, both clinical forms exhibit differences not only in terms of genetic background predisposing factors but also in their clinical presentations and the cerebral topographic pathology form (10, 11). Moreover, clinical, neuropathological, and molecular commonalities are also described to bridge AD clinical forms [for review, see (12)].

AD is now recognized as to be initiated decades before the clinical symptoms are evident as recently reviewed (13). Mild cognitive decline is an early indicator of pathology. It is therefore important to evaluate systematically factors that might be causal effects or accelerators of this pathogenic process.

In this direction, epidemiological studies support the association between cognitive dysfunction and diabetes (14). It is likely that the “apparently trivial” glycemia or the ensued peripheral insulin levels somewhat impact on the brain cognitive function (diabetes duration, blood glucose levels, the rate of glucose tolerance, and the levels of glycated hemoglobin) all correlate with cognitive functional performance (15, 16). This has led some to consider dementia as a new form of type 2 diabetes mellitus (T2DM) complication (17), and although AD can occur independent to diabetes, the latter is considered a *bona fide* risk factor for dementia and AD (18–20). Accordingly, a meta-analysis of 28 studies concluded that in the diabetic population, the risk for all types of dementia is increased by 73% (21).

Despite the prolonged efforts invested in disentangling cerebral energy metabolism, critical aspects remain to be clarified, especially in relation to energy precursors' consumption and metabolism. Similarly, controversies exist in relation to the hierarchy order within the pathophysiological cascade for the roles of Aβ and mitochondrial dysfunction. Nevertheless, it is beyond the scope of this article to resolve these controversies. Herewith, we aim to review some aspects of the current knowledge of the exciting field of AD pathophysiology, focusing on reports that point to insulin signaling in the central nervous system (CNS) as a novel and promising pathogenic and therapeutic field of inquiry. We outline the nosogenic involvement of IR as a primary trigger with three interrelated factors: (1) neuroinflammation, (2) the pathogenic role of Aβ and p-Tau, and (3) mitochondrial dysfunction in the form of interdependent and self-perpetuative cascades. The literature search for this manuscript included Medline/PubMed, Google Scholar, Scielo, and Bioline International (www.bioline.org.br) data sources. Online English literature was searched using search terms for conceptualization of AD.

## Brain Insulin Axis Physiology

The physiological spectrum of insulin is broad while playing a definitive anabolic role by positively regulating glucose, proteins, and fat metabolism (22). Importantly, this hormone is thought to play a critical role in mitochondrial biogenesis (23), which denotes its biological and evolutionary connotation.

Insulin mediates its physiological actions through binding to tyrosine kinase activity receptors. The complex biology of the insulin receptor signaling transduction regulation has been previously documented (24, 25). The tyrosine autophosphorylation allows for the gain-of-function of the receptors and the ensued downstream phosphorylation of other substrate proteins identified as insulin response substrates (IRSs) 1 to 4 in mammals. The tissue-specific expression and differential binding of downstream signaling proteins dictate a particular and compartmental pattern of physiological actions (26). Phosphorylated IRS proteins bind and activate catalytic enzymes as phosphatidylinositol 3-kinase (PI3K) and a type of phosphotyrosine phosphatase. PI3K controls metabolic events as the translocation of glucose transporter proteins, glycogen, lipid and protein synthesis, anti-lipolysis, and the control of hepatic gluconeogenesis. Aside from PI3K, the RAS/mitogen-activated protein kinase (MAPK) cascade is involved in the anabolic, mitogenic, and pro-hypertrophic actions of insulin (27, 28). Furthermore, activation of these pathways improves learning and memory (29), stimulates neuronal growth, and enhances neuronal survival (30).

Insulin is a key factor modulating the destiny of neuronal stem cells in neurogenic niches. Insulin and insulin-like growth factor type I (IGF-I) signaling pathways promote neurogenesis by modulating stem cell proliferation, differentiation, and survival (31). Contrariwise, abnormalities in insulin signaling leading to impaired glucose metabolism or reduced glucose input into the brain may influence the course of brain aging (32, 33). IRS-1 and IRS-2 are major targets of inhibitory signals when more than 50 serine/threonine residues become phosphorylated, thus eventually leading to functional neutralization of catalytic tyrosine. A sort of feedback of serine/threonine phosphorylation of IRS underlies the magnitude of insulin stimulation (34). Therefore, an intricate balance between IRS phosphorylation at serine or tyrosine residues determines the extent and magnitude of insulin actions (35).

The regulation of glucose transporters by the action of insulin is endowed with an organ specificity pattern or local compartmentalization. Accordingly, neuronal glucose uptake and utilization are only influenced by glucose transporter 3 (GLUT-3), which is co-expressed with glucose transporter 4 (GLUT-4). Remarkably, the brain can metabolize glucose independent of any insulin action given that glucose can simply diffuse across the blood–brain barrier (BBB) (36).

Most brain insulin derives from the systemic circulating pancreatic insulin, transported into the brain where it enters by means of a selective and saturable carrier on capillary endothelial cells of the BBB (17). Compelling evidences document that the brain is definitively an “insulin sensitive organ” (37) and diverse methodological approaches indicate insulin *de novo* synthesis in the brain [for review, see (38)]. In line with this, physiologically active insulin receptors and their signal transduction pathways have been localized in several regions of the brain, intervening in a broad spectrum of neurophysiological actions such as attention, executive functioning, learning, and memory (38). Furthermore, hypothalamic insulin also regulates hepatic glucose production (39), emphasizing that central insulin establishes a functional connection to peripheral organs with systemic repercussion.

Brain glucose metabolic fate is largely impinged by the specific cell stripe and its metabolic demands. Exemplarily, neurons exhibit an oxidative metabolism while astrocytes are mostly glycolytic (40). Although brain energetic homeostasis alterations may influence the ignition and progression of various neurodegenerative disorders in which glucose metabolic rates decline (40), the links within the pathogenic cascade, as the precise role played by the different cerebral cell populations, remains to be clarified.

## Central Insulin Resistance

Since decreased brain insulin levels or insulin receptor signaling is associated with impaired cognitive functions and neurodegenerative diseases, the mechanisms involved in central insulin signaling, glucose utilization, and neuronal energetic homeostasis are currently emerging as a promising research/interventional area (41–46).

Brain IR is simply defined as a state of failure of brain cells to respond to insulin input (47), which may extend to reductions in both the levels and signaling of IGF-I and IGF-II (48). Although this lack of response to insulin could mechanistically respond to reduced neuronal transcription or low protein expression of insulin (49–51), its receptor (43, 49, 52), the receptor substrates (49), and to an interference in receptors tyrosine-kinase activity (43, 53), the latter appears as a *sine qua non*.

As defined by Kandimalla et al., insulin cerebral physiology deserves far more attention given the broad physiological implications and pathophysiological repercussions of this “metabolic hormone” in the CNS homeostasis (54). This is simply attested by clinical evidences that document neurodegeneration and cognitive deterioration in hyperinsulinemic/insulin-resistant subjects even under normal glycemic levels (55) and by the experimental demonstrations of insulin receptor derangement and the ensued catastrophic neuronal starvation upon streptozotocin (STZ) intracerebroventricular administration (56). We and others share the view that insulin axis failure and the subsequent glucose hypometabolism are reminiscent of neuronal pro-senescence traits and accordingly a cerebral pro-aging condition. Thus, LOAD may represent a particular form of “uncompensated” (57), precocious, and pathologic organ-specific senescence supported by a dysmetabolic base. As a matter of fact, IR, glucose hypometabolism, oxidative stress, mitochondrial dysfunction including PGC-1 underexpression, Aβ accumulation, mitochondrial and nuclear DNA damage, and cognitive decline are hallmarks of both cellular senescence and organismal aging (58). The molecular drivers bridging cerebral aging with AD initiation and progression were exhaustively reviewed by Mao and Reddy (59). Yet this is an area of intense debate and investigation given the fact that some studies demonstrate that Aβ deposition is not a *sine qua non* for hypometabolism and cognitive decline (60, 61).

Returning to insulin discussion, as excellently reviewed by Kandimalla et al. (54), its role in the brain goes far beyond the control of glucose uptake and utilization for energetic purposes. The hormone is endowed with pro-survival, trophic, and anti-apoptotic effects, thus promoting neurite growth and axonal regeneration. Via the agonistic stimulation of the PI3K/Akt/GSK-3β pathway, insulin prevents Aβ intraneuronal accumulation and modulates Tau metabolism. Of note, insulin is also bestowed with an anti-inflammatory effect that may even attenuate the hyperglycemia-mediated inflammation (62, 63). In general terms, insulin along with the broad intracerebral distribution of its receptor may act as a sort of cerebral safeguard for global neuronal physiology and healthy mental processes like behavior, emotions, cognition, learning, and memory (64–69).

**Figure 1:**
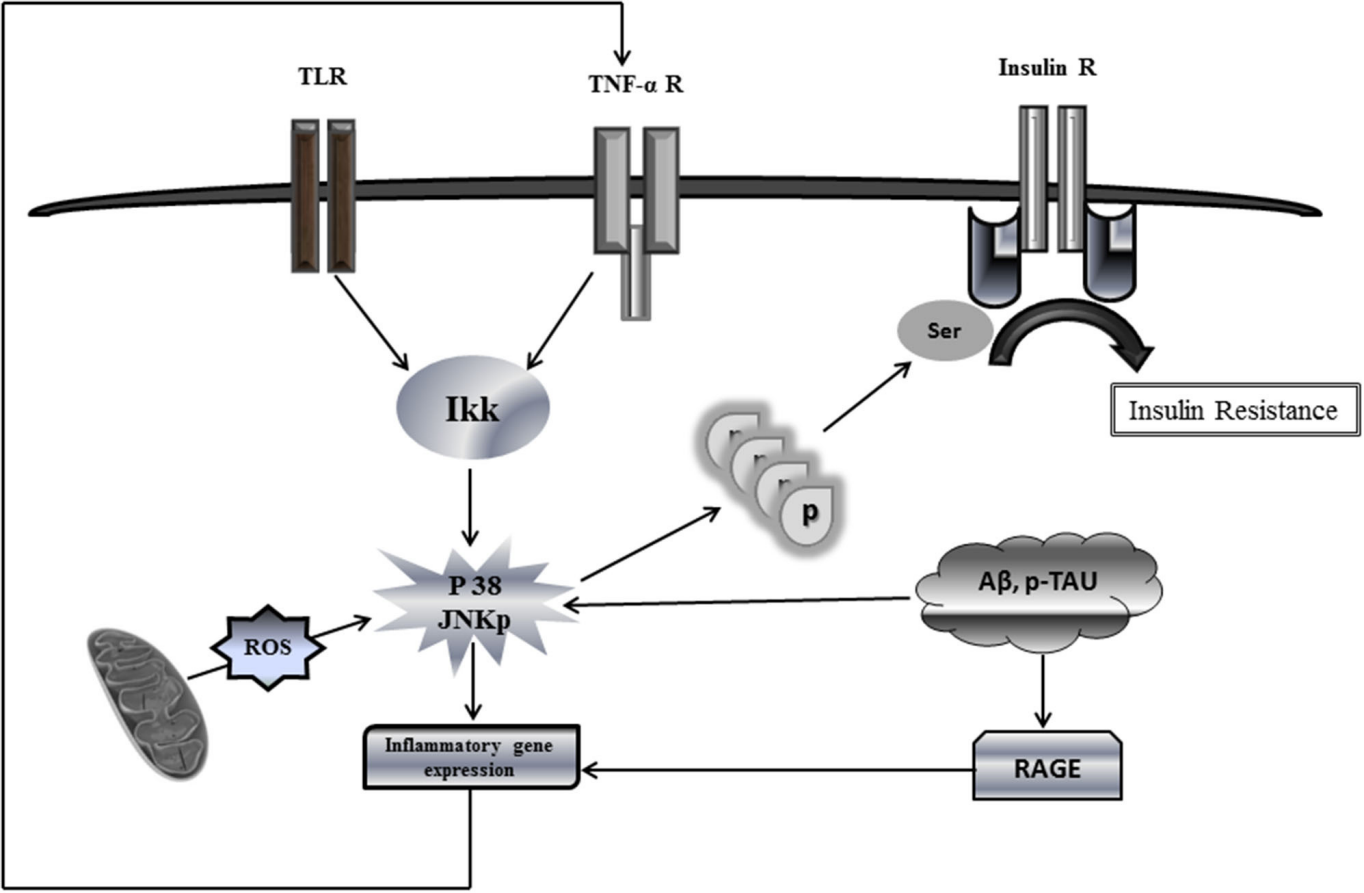
Putative mechanistic model of insulin resistance via c-Jun N-terminal kinase (JNK) activation. This simplified putative model describes JNK activation pathway as a phosphorylation target of a variety of extracellular stimuli, e.g., proinflammatory cytokines as well as intracellular stimuli, oxidative stress, and oxidized mtDNA. Upon activation, phosphorylated JNK promotes direct serine phosphorylation of insulin receptor substrate protein IRS1, thus causing a defective IRS1 tyrosine phosphorylation and reduced phosphatidylinositide 3 kinase (PI3K) and AKT signaling in response to insulin receptor activation. Phosphorylation of serine residues inhibits the interaction of IRS1 with the insulin receptor, thereby blocking the response to insulin. Being applicable this model for neuronal IR would lead to disastrous consequences for most of the cellular stirpes within the brain. JNK activation by ROS-free radicals, Aβ, and hyperphosphorylated Tau promotes the transcriptional expression of proinflammatory cytokines, which in turn may enhance the production of ROS, the mitochondrial dysfunction, and the accumulation of neurotoxic Aβ+p-Tau, which ultimately enhance IR. Similarly, RAGE activates the transcription of proinflammatory products. A putative vicious circle would presuppose that ROS spillover within the mitochondrial environment amplifies inflammation, impairs, and damages OXPHOS enzymes and provoke mtDNA damages/mutations. These events ultimately synergize and amplify IR and neuronal energetic collapse, leading to the organelle fragmentation. Accordingly, the pool of dysfunctional and fragmented mitochondria will induce neuronal demise.

Among the mechanisms analogous shared by T2DM and AD, it is the peripheral impaired insulin signaling that may account for brain IR in AD (9). Members of the c-Jun N-terminal kinase (JNK) family of MAPK have recently emerged as important players in AD, not only because of their increased phosphorylated expression in human postmortem brain samples and its positive co-localization with Aβ (70) but also because of their role in mediating degeneration and apoptosis in the brain, not to mention its hindrance over the neuronal insulin axis physiology. AD assembles multiple stressor factors that are known to activate JNK pathway as oxidative stress, Aβ accumulation, neurotrophic deprivation, and proinflammatory cytokines such as tumor necrosis factor alpha (TNF-α) (71). JNK activation, driven by either free radicals or proinflammatory cytokines, results in IRS-1 serine phosphorylation (Figure 1) blocking downstream insulin signaling. The ensued IR entails not only a glucose-related energetic dyshomeostasis but also the central depletion of one of the major neurotrophic factors, which account for neuronal vulnerability and the amplification of JNK pathway activation.

Hegde et al. (72) have enriched the concept that hyperinsulinemia and IR are integral pieces of the AD pathophysiology conundrum and that hierarchically speaking, the latter may simply be a consequence of the former (73). Above all, these insulin impairments collectively translate in the collapse of glucose homeostasis leading to cerebral hypometabolism, neuronal damage, and cognitive deterioration (72). Foundational (74) and subsequent studies [for review, see (75)] have documented the remarkable variety of biochemical outcomes associated with the interaction of insulin with its receptors. Nonetheless, chronic exposure to insulin or to high insulin levels, globally speaking, desensitizes the receptor, blocking the response to insulin (73). Addressing this problem within the AD pathology, it is known that peripheral hyperinsulinemia is followed by an impaired signaling process within the brain (76), which accounts for the subsequent activation (dephosphorylated form) of GSK-3β, thus amplifying IR, amyloid β accumulation, and hyperphosphorylated Tau accumulation (77, 78). Conclusively, neuronal starvation, bioenergetic failure, and neurotoxic amyloid accumulation may be among the primary consequences of hyperinsulinemia-mediated impairments via IR.

Multiple experimental approaches involving the aforementioned AD-related factors rendered convincing evidences supporting the concept that JNK activation may account for cerebral IR [reviewed in (9)]. In line with these evidences, mitochondrial dysfunction has also emerged as an essential research target of the AD neuronal energetic collapse (32) as discussed below. Irrespective to what may look controversial across the history of findings, inhibition of tyrosine residues phosphorylation may signify a hierarchal event for the insulin receptor “loss-of-function” phenotype (79), which may play a crucial pathogenic role in AD (80) and account for a neurodegenerative process.

Despite the differences in the origin of the disorders, in their proximal triggers, as in the individual risk factors for the two most important clinical forms of AD, both eventually deteriorate patients' cognitive and behavioral abilities in a similar manner (81). Yet it is likely that IR holds a different chronological and hierarchal position in the pathogenicity tree for each of the forms. We therefore share the Correia notion that IR remains as the most notable and primary nosogenic candidate within the core of the LOAD neurodegenerative cascade (82). Simply said, from the IR master switch position, a downstream pathophysiological cascade may be turned on, eliciting irreversible and unstoppable degenerative events, via secondary drivers as Aβ, Tau, and oxidative stress. All these drivers are integrated in perpetuative and forward loops (83). For the familiar form, however, we point to its numerous mutations that convergently yield errors in APP proteolytic processing, rendering neurotoxic Aβ products that, in synergy with Tau, trigger a downstream series of neurodegenerative events in which inflammation, oxidative, and IR cooperatively participate [for review, see (84)].

Since LOAD is considered by some as type 3 diabetes (85), and given that metabolic memory is the steering wheel toward irreversible multiorgan diabetic complications (86), including a progressive cognitive decline (16), it is alluring to examine how diabetes-like epigenetic-derived signatures are implicated in the perpetuation of the multiple pathogenic events of AD. More precisely, are the molecular drivers of metabolic memory acting in extracranial structures (87) pathogenically involved in central IR-associated neurodegenerative diseases? As smartly poised by de Felice et al., an assortment of challenging pieces still wait for clarification: (1) How does peripheral IR impact brain metabolism? (2) How do peripheral insulin level oscillations impact cerebral cells metabolism? (3) How do peripheral IR-derived metabolic disorders progressively yet silently impact the brain? (4) Is there a brain neuron subpopulation intrinsically more susceptible to develop LOAD or any other form of dementia upon peripheral IR? (88).

## Major Nosogenic Mechanisms

Without the ambitious expectancy to discern a sequential hierarchic pathogenic responsibility, and following the conception of AD as a primary organ-specific, diabetes-like disease, we review the pathogenic involvement of central inflammation, neurotoxin accumulation, and mitochondrial dysfunction as critical nosogenic drivers adjacent to IR/insulin dysfunction and brain energetic dyshomeostasis.

### Role of Inflammation

A wealth of evidences have emerged to link peripheral (89) and central inflammation with IR, cerebral energy hypometabolism, and neurodegenerative diseases, including AD (67, 90, 91). Conceptually, the brain is susceptible to a potential double hit interconnected inflammatory loop: a centrally originated neuroinflammatory response; and a systemic, peripheral cytokine storm that may invade the brain parenchyma once the BBB is breached. Diverse studies highlight elevated proinflammatory cytokines, chemokines, acute phase reactants, and other inflammatory molecules in the circulation of AD patients, with some of these cytokines as interleukin-6 (IL-6), positively associated with cognitive decline and AD progression (92). Moreover, in this context, IR is a constant phenotype. Accordingly, this cytokine-induced IR is derived from neuronal insulin signaling deficiency, which is ensued by mitochondrial functional demise, thereby originating a vicious circle inflammation-oxidative stress that catalyzes neuronal energy failure (32, 69).

**Figure 2:**
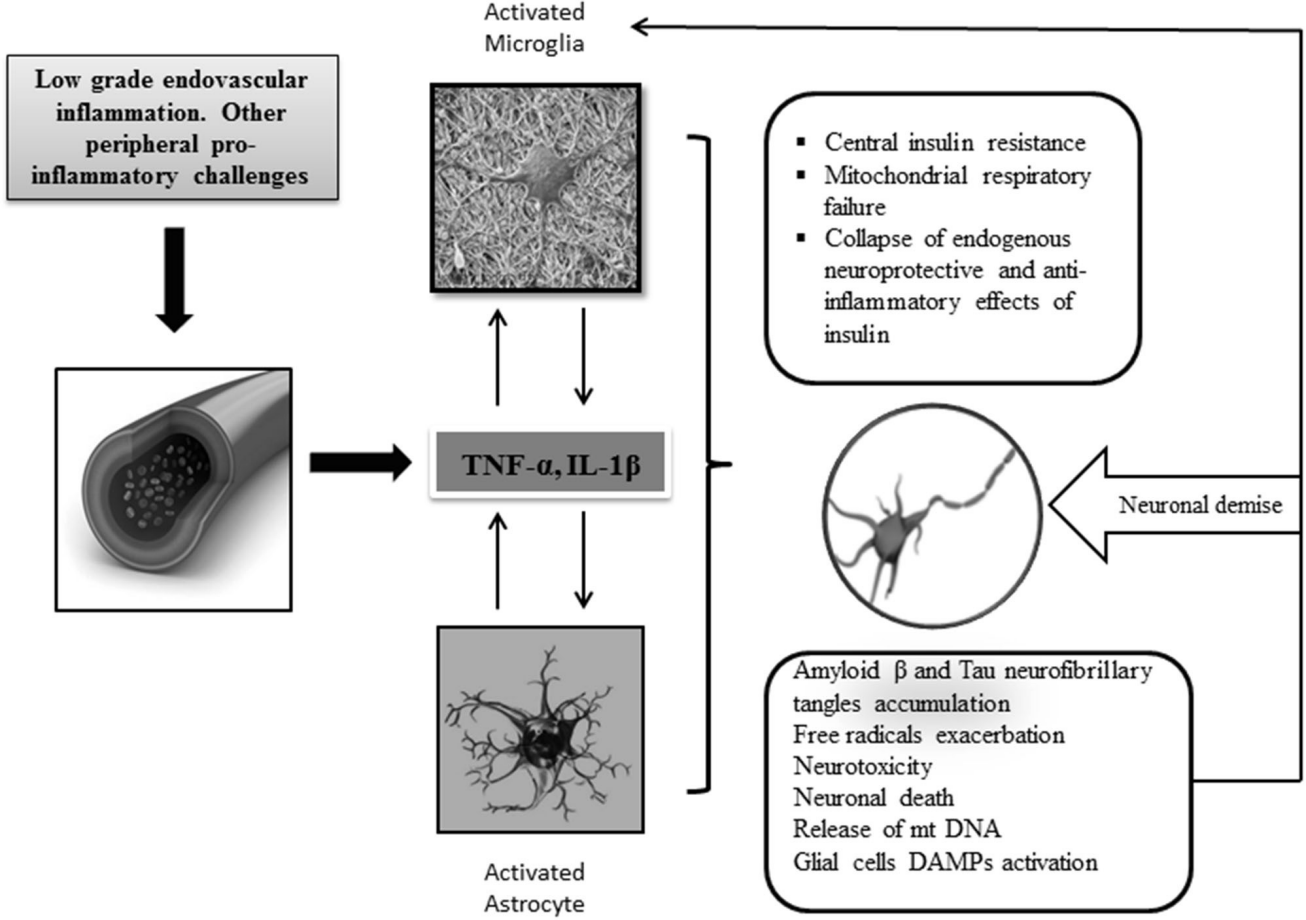
Central neuroinflammation and consequences. Increased levels of peripheral and CNS of proinflammatory mediators support the key role of inflammation in AD pathology. Priming of glial cells to mount a protracted proinflammatory phenotype is a critical event that seems linked to aging, circulating peripheral cytokines, ROS, and neurotoxic amyloid β accumulation. Cross talk between microglia and astrocytes leads to the generation of proinflammatory/neurotoxic astrocytes, which further enhance the production of inflammatory cytokines and chemokines leading to a detrimental gliosis and astrocytosis in which, for instance, central IR and neurotoxic β amyloid accumulation are intensified. Under these conditions, mitochondrial function is further impaired, and consequently, free radicals are over-produced. Dysfunctional mitochondrial clearance is impaired. The central proinflammatory environment accounts for the amplification of this series of interrelated events eventually leading to progressive energetic collapse and neuronal and synapsis loss. The inflammatory cascade may become intensified when glial cells are further activated by the presence of released DAMP ligands, for instance, oxidized mtDNA, neuronal sequestered antigens, and accumulated β amyloid. In general, the disease is perpetuated by the interconnection of different pathogenic vicious circles, which amplify each to another.

Cytokines drive neuroinflammation in every respect (Figure 2), (93). IL-1, IL-6, TNF-α, and transforming growth factor beta (TGF-β) are pathogenically involved in AD neuroinflammatory process, which entails insulin axis failure and, consequently, cerebral glucose hypometabolism. Although the expression of these cytokines is induced by the presence of Aβ peptide, they are also promote the accumulation of Aβ peptide. Altogether, these cytokines are considered as key players of a vicious circle perpetuating AD (94). The interconnected relationship involving central inflammation/cerebral glucose metabolism/cognition has been documented by the intracerebroventricular administration of STZ to otherwise normal animals (54). Intracerebroventricular injection of STZ is associated with an intense astrogliosis and neuronal inflammation, alterations of the brain insulin system, decrease in glucose utilization, oxidative stress, and ultimately progressive learning and memory deficit (95). Other animal studies have enriched the pathogenic link embracing inflammation-cognitive impairment. APP/PS1 double transgenic mice grafted with gut microbiota from AD patients exhibited intestinal elevation of NLRP3 inflammasome and systemic circulating inflammatory mediators. This peripheral inflammatory reaction with concomitant cognitive impairment was associated with activation of microglia in the central hippocampus and increased expression of local neuroinflammatory biomarkers (96).

Microglia, astroglia, and perivascular macrophages are major cellular levers of neuroinflammation via the innate immune system (97). These cells have a common characteristic of being largely glucose consumers that become reactive upon peripheral injury or systemic metabolic stress, thereby increasing the paracrine production of proinflammatory cytokines. “Microglial cells represent the immune system of the mammalian brain” and consequently are critically involved in various diseases. Activation of microglia is a hallmark of central inflammation and conceivably of brain pathology (98). Microglial cell reactivity, via the establishment of a local cytotoxic milieu, impairs neuronal homeostasis. Interestingly, single-cell transcriptomics studies showed that inflammatory gene expression can progressively switch according to the disease state and to the proximity of the Aβ (99). Again, early inflammatory activation by MAPK and the JAK-STAT pathways appeared in microglial cells even before Aβ deposition (100, 101).

Integrative mathematical models support the pathogenic role of microglial cell activation and reactivity. Models based on “differential rate equations” that represent cellular cross talks (involving microglia, astroglia, neurons, and Aβ) have indicated that the microglial inflammatory activation acts as a sort of “key node” for progressive neurodegeneration. Accordingly, the model proposes microglial reactivity as a potential target for the prevention and treatment of AD (102).

Soluble Aβ oligomers bind to microglia and fibrils via toll-like receptors (TLR2, TLR4, TLR6, and TLR9), which are essential components of the innate inflammatory cascade (103). Letiembre et al. offered the first evidence for a role of the key innate immune receptor TLR4 in neuroinflammation in AD by demonstrating that TLR4 contributes to Aβ peptide-induced microglial neurotoxicity (104). Activated microglia and hypertrophic reactive astrocytes accumulate around Aβ plaques, as observed in postmortem human AD tissue (105) as in animal models (106). Persistently activated microglia releases proinflammatory cytokines that contribute to exacerbate Aβ and/or Tau-associated pathology (107). Microglial cells neighboring Aβ plaques in AD patient brains are a source of IL-1β, which is in correspondence with the *in vitro* finding that the cytokine appears to be released by activated microglia after stimulation with Aβ (108). Furthermore, microglial cell-derived IL-1β favors astrocyte activation and astrocytic overexpression of S100B and Aβ expression and deposition by modulating APP expression and proteolysis (109, 110).

Activated microglia has been found near neurofibrillary tangle (NFT)-bearing neurons (111). Phagocytosed Tau induces inflammasome activation inside microglia, causing overactive microglial state, which could be one of the mechanisms that promote the constant inflammatory response in AD (112).

Another pathogenic stream links a failure of the mitophagy process with the onset of a central inflammatory reaction. As described by Fang et al., a failure in purging defective mitochondria plays a definitive pathogenic role in AD. Hence, mitophagy poises as a potential therapeutic intervention to ameliorate neurodegenerative diseases via reduction of inflammation (113). Different observations suggest that during chronic neuroinflammation, microglia become incompetent for Aβ plaque clearance (114). Consequently, induction of mitophagy mechanisms to clear diseased and Aβ-overloaded mitochondria reduces neuroinflammation (114).

Increased levels of microglia-derived cytokines as interferon gamma (INF-γ), IL-1β, IL-6, and TNF-α induce astrocytes to adopt a classical activation state (115), being predominantly subclass A1 astrocytes. Although glial activation can occur independent of Aβ stimulus, it is likely that interaction of Aβ with astrocytes is largely responsible for the induction of a pro-inflammatory profile and astrogliosis (116). Under these circumstances, the beneficial properties of astrocytes are lost given the continuous reactive gliosis and the brain injury propagation (91). Astrocytes may therefore fail in Aβ plaque phagocytosis and secretion of different Aβ-degrading proteases, which may increase amyloid pathology, inflammation, and neuritic atrophy (117). Astroglia atrophy may have far-reaching consequences for synaptic connectivity, thereby contributing to cognitive deficits (118). Activated astrocytes produce TNF-α, which amplifies inflammatory demyelination. These astrocyte-induced proinflammatory mediators have shown to produce synaptic disturbances and neuritic dystrophy in different AD mouse models. The astrocyte-derived proinflammatory mediators may ultimately amplify Aβ pathology by alterations in the homeostasis of APP processing (119). Astrocytes are also implicated in calcium extra-synaptic glutamate receptor activation and glutamate excitotoxicity, which accounts for the generation of reactive oxidative species and neuronal oxidative stress (120). Again, Figure 2 outlines the main damage pathways of neuroinflammation.

Of paramount significance is that most of these inflammatory mediators activate the JNK pathway, which definitively contributes to uncouple insulin signaling (121). Mice fed with polyphenolic anti-inflammatory diet agents exhibited an ameliorated cognitive performance (122), which emphasizes the pathogenic role of neuroinflammation in cognitive degeneration. An exciting work provided by Clarke et al. showed that intracerebroventricular administration of AD-associated Aβ oligomers in mice elicited peripheral glucose intolerance. Conversely, blockade of the Aβ and the endoplasmic reticulum (ER) stress reduced neuroinflammation and attenuated peripheral glucose intolerance. These findings indicate that peripheral tissue glucose resistance is centrally evoked by Aβ oligomers (123). The oligomer-induced neuronal IR is mediated by TNF-α activation via JNK pathway with the ensued insulin receptor inhibition, which has major negative impact on synaptic function, synaptic plasticity, and synaptic connectivity (124–127). This TNF-α counter-insulinic effect, in which the insulin receptor becomes inactivated by increasing the inhibitory phosphorylation threshold of the IRS1, has been broadly described in diabetes (128) and in neurons presenting Tau-pathology and NFT (129). These IRS inhibitory phosphorylations described in AD patients' brains and transgenic mice lead to IR states (34), memory declines, and cognitive impairment (130).

The multiligand receptor for advanced glycation end products (RAGE), member of the immunoglobulin superfamily and able to bind a heterogeneous group of ligands, is involved in a horizon of physiological settings and pathological realms. RAGE therefore is a pathogenic leverage in proinflammatory, oxidative, and degenerative processes like AD (131). Increased RAGE immunoreaction has been detected on microglia and neurons of the hippocampus, entorhinal cortex, and superior frontal gyrus from AD individuals.

RAGE agonistic stimulation by the cognate ligands is associated with proinflammatory and prothrombotic events with the ensued upregulation of chemokines, free radicals, and proinflammatory cytokines (132). Of major significance for AD neurodegeneration pathology is that RAGE binds Aβ peptide with high affinity, which is supported by its ability to rapidly transport brain-derived Aβ into the peripheral circulation (132). The noxious duet of Aβ-RAGE develops a positive forward circuitry in which microglial activation enhances the expression of macrophage colony-stimulating factor (M-CSF) and RAGE, possibly triggering a spiral of cellular activation (133). Ghidoni et al. have documented reduced levels of circulating soluble RAGE in patients with mild cognitive impairment. Soluble RAGE could therefore prevent Aβ neurotoxicity and stimulate Aβ clearance from the brain, consistently accentuating the role of the RAGE axis in AD pathogenesis (134).

Conclusively, neuroinflammation stands as a crucial pathogenic driver in the pathogenesis of AD. The intricate cascade of both central and potential peripheral inflammation accounts for the inability of the insulin receptor signaling, which in turn leads to neuronal energy dyshomeostasis. The activation of JNK pathway is likely at the crossroad between inflammation and insulin axis impairment. The central gliosis generated further amplifies inflammation with the ensued consequences of IR, energy dyshomeostasis, oxidative damage, and finally the eventual neuronal bankruptcy. Central inflammation is also linked to mitochondrial dysfunction; accumulation of neurotoxic amyloid material, which obviously increases mitochondrial functional demise, DNA, and protein oxidative damage; and a failure of glial cells in purging the environment of wrecked mitochondria (mitophagy).

### Amyloid Beta, Hyperphosphorylated Tau, and Insulin Resistance in Alzheimer Disease

Aβ peptide 42 (Aβ42), Aβ40, and Tau phosphorylated at threonine-18 are considered active nosogenic players in the core of AD pathology. Accordingly, the deposition of extracellular aggregated Aβ, along with hyperphosphorylated Tau, and intracellular NFTs are identified as histopathological hallmarks of AD (135, 136).

The perilous combination of Aβ and hyperphosphorylated Tau, directly and indirectly, causes a complex and ample neurotoxic damage explosion that impairs chemical neurotransmission, axonal transport, and ATP synthesis-energy availability, eventually concluding in synaptic loss and cognitive deterioration (137). The parenchymal accumulation of amyloidogenic material is perhaps the most distinguishing histopathological feature of AD and the base of its clinical expression (138). Aβ derives as a by-product of an aberrant processing of APP by two main enzymes: β and γ secretases; moreover, native APP appears to act in facilitating the processes of memory and learning via synapses and dendritic spine formation (139). Aβ peptide derived from the proteolytic processing is basically released to the extracellular space in health conditions during neuronal activity, whereas its levels are controlled by local proteases. Errors in the cleavage position may lead to the increase of the Aβ1–42 neurotoxic isoform, which constitutes aggregates that spread the parenchymal neuropathological damage (140). Neurotoxic Aβ intracerebral administration is associated with a memory deficit reminiscent to that observed in AD patients. These memory and learning deficits are related to synaptic plasticity disruption via the intraneuronal accumulation of Aβ (141). A parallel lane of Aβ-mediated neuronal damage is related to the immune-inflammatory activation triggered in glial cells leading to neuronal and synaptic structure phagocytosis (142, 143). Thus, Aβ intraneuronal accumulation is poised as a central driver for a variety of synaptopathies.

Aβ metabolism is impacted by insulin and the threshold of insulin receptor sensitivity, whereas, conversely, Aβ interferes with insulin binding to its receptor and the expected biological response. To briefly exemplify this assertion, a failure in insulin receptor and/or its accessory substrates proteins (IRS) aborts A*kt* phosphorylation as the downstream inactivating phosphorylation of GSK-3β. Accordingly, this non-phosphorylated state of GSK-3β exhibits proinflammatory effects, obstructs glucose clearance, increases the accumulation of Aβ via presenilins, and promotes Tau phosphorylation within NFTs [for review, see (144)]. Contrariwise, insulin contributes to the extracellular excretion of Aβ and to its enzymatic degradation via the insulin-degrading enzyme (IDE) (145). Nevertheless, insulin *per se* is a preferential substrate for IDE acting as a competitive inhibitor for the enzyme, thereby indirectly promoting Aβ accumulation (146). Of note, autopsy processing of AD-derived brain samples has shown a reduction of the IDE activity, which was considered a meaningful risk factor for AD (147).

Interestingly, Aβ oligomers that accumulate inside cells can subsequently spread its pathologic message to normal healthy neurons via exosomes, promoting a variety of neurodegenerative changes (148). This finding implies that exosomes may act as potent, soluble pathology vectors in AD and offers the opportunity to investigate its potential pathogenic similitude with the role played by the senescence major messenger, the so-called “senescence-associated secretory phenotype” (SASP) (149). Of note, metabolic dysfunction is an organismal driving force for aging and a senescence hallmark (150), with meaningful repercussion in diabetes via the vicious cycle embracing mitochondrial/metabolic dysfunction + free radicals spillover + telomeric integrity (151). These disperse pathophysiology pieces represent a constellation of commonalities between T2DM and AD.

In pathogenic tandem to Aβ is Tau, which is responsible for neurodegenerative events by the production of NFTs, which ultimately harm neurons and synaptic connections (152). Neuronal microtubules and cytoskeletal assembly are largely accomplished by Tau proteins (153). Having said that, it is inferable that abnormalities in Tau-cytoskeletal system bring about filament aggregation, synaptic transmission failure, and ultimately neuronal demise (137). Tau protein hyperphosphorylation disassembles microtubules and elicits a variety of cytoplasmic and axonal damages that conclude in neuronal death (154). Tracking the regional pattern of Tau-induced cerebral damages suggests that its pathology disseminates in a prion-like manner, ultimately leading to progressive cognitive deterioration (155). Tau hyperphosphorylation is also a collateral consequence of cerebral IR (156), whereas insulin oligomers are retained by hyperphosphorylated Tau (157), thus hampering insulin activity.

Although the amyloid accumulation and the Tau hyperphosphorylation have nurtured classic pathogenic AD hypothesis, the identification of a primary mechanistic connection and a nosogenic hierarchic order between Aβ and Tau has been difficult so far (158, 159). Nonetheless, they appear to act together in an inter-perpetuative manner. Aβ and Tau toxicities are linked stressors in the core of AD pathophysiology as shown by transgenic mouse models, which poise Tau as an additional force, amplifying Aβ pathogenicity within the postsynaptic environment and the dendritic spines (160). Furthermore, Aβ oligomers from AD brain extracts increase Tau phosphorylation, whereas the mechanisms mediating this hyperphosphorylation state drive to Aβ accumulation (161, 162). They both eventually disturb the insulin axis function, leading to IR and further Aβ and Tau-mediated pathology (163). Again, the onset of a vicious circle is established between the insulin signaling system and these two synergistic neurotoxic ingredients.

Although Tau localization from the soma to the dendrite can be influenced and modified by intrinsic and extracellular factors, Tau pathology is compellingly associated with cognitive impairment in AD and other forms of dementia [for review, see (164)]. Having summarized general elements of the Aβ and the hyperphosphorylated Tau in a rather simplistic manner, we will briefly outline their pathogenic involvement in neuronal synapses and neurotransmission (Table 1). The foremost concepts of Aβ and hyperphosphorylated Tau in synaptic and neurotransmission pathology were comprehensively and exquisitely reviewed by Rajmohan and Reddy (137).

**Table 1:** Summarized synaptic damages associated with Aβ toxicity and hyperphosphorylated Tau.

	**Amyloid β**	**Hyperphosphorylated Tau**
Synaptic structural and functional damages	◦ Synaptic plasticity impairment (165, 166). ◦ Synaptotoxic effect (167) with ultrastructural synaptic damage. ◦ Synaptic loss, destruction of axons, and dendrites (168). ◦ Synaptic transmission failure (169). ◦ Neuronal hyper excitability and eventual death (170).	➢ Mediates Aβ postsynaptic and dendrites toxicity/damage (160). ➢ Accumulates and harms dendritic compartments (171). ➢ Impairs glutamate receptors, synaptic trafficking, and postsynaptic physiology. Ultimate neuronal degeneration/death (172).

*Here is a summary of the major damages brought about A-beta and p-Tau*.

That (1) synaptic and dendritic damages and neurotransmission alterations are underlying forces driving to loss of connectivity, memory deficit, cognitive impairment, and dementia in general (137, 173, 174); (2) large experimental data have been accrued the relevance of insulin axis for the neuronal physiology and survival (6); and (3) the tremendously negative impact of brain insulin axis bankruptcy in aging and AD brains (6, 175, 176) judiciously vindicate insulin interventions as an avenue to mitigate AD progression and ultimately reduce cognitive impairment. Converging data from animal models indicated that insulin treatment attenuated hypometabolism, improved spatial memory, reduced inflammation, and significantly diminished amyloidogenic accumulation and Tau hyperphosphorylation (177, 178). These data are validated by proof-of-concept studies that suggest an improvement in brain glucose metabolism, as in the spheres of memory and cognition (179–181).

Taken together, these data reinforce the notion that a finely and steadily controlled tuning of the insulin axis is crucial for normal neuronal metabolism and accordingly of amyloid and Tau processing, recycling, and disposal. Central and/or peripheral factors leading to failures in the insulin axis control system with the subsequent aberrations in glucose processing may be sufficient to elicit other nosogenic effectors in AD (137).

### The Role of Mitochondria

More than 90% of the body's cellular energy is generated in mitochondria by oxidative phosphorylation. Concomitantly, mitochondria are also the major manufacturing plant of reactive oxygen species (ROS) via the electron transport chain. Therefore, ATP and its by-product ROS are crucial performers in most physiological and pathological processes, which unquestionably emphasize the evolutionary significance of the organelle (182, 183). Mitochondria are responsible for broad regulatory functions in embryonic and postnatal life in a full spectrum of cells, tissues, and organs via oxidative balance, calcium homeostasis, and programmed cell death (184).

As already mentioned, brain IR prevails in AD and other neurodegenerative conditions via insulin receptor desensitization through IRS1 inhibitory phosphorylation (185). In this scenario, mitochondrial dysfunction in neurodegenerative pathologies is not a fortuitous event. Mounting evidences have begun to elucidate what the missing links are, connecting IR and mitochondrial failure in cerebral (186) and extracerebral tissues (187). Illustratively, overexpression of PGC-1α, the master controller of mitochondrial biogenesis and metabolism (188), rescues both insulin signaling and mitochondrial bioenergetics in skeletal muscle cells, poising it as the link between insulin axis functions and mitochondrial homeostasis (189). PGC-1α levels are found to be in a negative balance in the brains of patients with neurodegenerative conditions, which is reasonably ensued by mitochondrial dysfunction and oxidative stress (190, 191). The biological preservation of PGC-1α expression has been invoked as a preventive factor for the generation of Aβ peptides (192) as to ameliorate neuronal loss and improve neurological symptoms (193). Whether PGC-1α activation may prophylactically prevent the metabolic havocs mediated by JNK activation pathway in neuronal cells remains to be examined.

Thus, the concept of brain hypometabolism encircles the insulin axis physiology and the complete set of mitochondrial oxidative operations. Hypometabolism is a mitochondria-related event (194).

Although the brain is only 2% of total body mass, this organ operates with 20% of the oxygen and 25% of the glucose economy, thus demonstrating its high-energy requirements (195). Neurons are broadly dependent on mitochondrial activity and energy production (195). Furthermore, neuronal energetic homeostasis depends upon functional mitochondria to cope with their high-energy demands, especially for synaptic processes. Contrariwise cerebral mitochondria functional collapse impairs memory function and translates in an ensemble of degenerative events (32). The pathogenic role of mitochondria is so clinically meaningful that it has been stated that its damage intensity and extension somewhat correlate with the course of the disease (196). Accordingly, mitochondrial functional impairment and energy dysmetabolism foster cognitive and working memory deterioration (197). Growing evidence demonstrates that maintaining mitochondrial bioenergetic function could prevent these age-dependent alterations (198).

Mitochondria are also committed to provide the fuel for a variety of ATP-dependent neuronal processes encompassing from synaptic transmission to neurotransmitter reuptake. Conversely, reduction of mitochondrial performance in ATP synthesis is the core of the molecular havoc that spans from calcium and synapsis dyshomeostasis to neuronal apoptosis (196, 199).

The underlying pathogenic involvement of mitochondrial dysfunction has been extensively documented in AD, including both the early-onset familial and late-onset sporadic forms of the disease (196, 200, 201). A collection of exciting findings shed light on the pathogenic involvement of mitochondrial dysfunction in age-related neurodegenerative processes, AD pathology and Parkinson's disease (202, 203).

Brain ultrastructural electron microscopy images dated more than four decades ago provided the inaugural evidences of mitochondrial morphological abnormalities in AD. Thus, the primary differences on mitochondrial structures were established for AD subjects and healthy-age-matched control subjects. The ultrastructural pathology of these mitochondria describes the organelle as smaller or dilated, shrunken and fragmented and with misshapen broken cristae. Overall mitochondrial size appeared reduced (204). Other studies indicated similar ultrastructural modifications in AD's brain vulnerable neurons in terms of significant size reduction and other alterations indicative of mitochondrial dynamics faults (205). In line with this, an aberrant distribution of mitochondria was found in pyramidal neurons of AD-affected individuals where mitochondria appeared redistributed away from axons (206).

Above all, a major pathogenic mainstay for LOAD-affected brain resides in fuel deficiency, particularly for glucose (207). Fluorodeoxyglucose positron emission tomography (FDG-PET) data definitively documented that there was a deficit in glucose utilization by the brains of AD patients. Abnormal cerebral glucose hypometabolism is a high sensitivity and a high specificity indicative of AD.

Albeit evidences that hypometabolism can be detected and predict at-risk individuals years before the symptoms onset, it remains elusive which are the etiopathogenic drivers underlying this pervasive process (208, 209). Thus, there is a subsisting debate on “who-precedes-who” between glycolysis impairment and mitochondrial dysfunction. The solid truth is that there is an underlying defect in cerebral glucose uptake and subsequent metabolism (210). Seminal PET studies indicated that cerebral glucose uptake and its subsequent transformation to glucose-6-phosphate by hexokinase is deficitary in AD (211). It was subsequently shown that several other glycolytic enzymes are also perturbed since the very early pre-symptomatic stages of AD (212), which seems to stimulate a cerebral metabolic reprogramming in search for other energy sources as ketone bodies to overcome glucose hypometabolism (207). Nonetheless, recent outcomes have indicated the existence of an initial stage of reactive cerebral glucose hypermetabolism prior to hypometabolism, for AD and other neurologic disorders in brain-specific areas (213).

Another neuronal hypometabolism arista is based on the negative consequence of the primary hyperinsulinemic spikes with resulting brain functional implications. Hyperinsulinemia, regardless of stimulating peripheral lactate production, hampers its neuronal availability and utilization by limiting its flux across the BBB (214). Once again, converging evidences portray how prolonged exposure to hyperinsulinemia downregulates the insulin receptor in critical cerebral cell lineages as glial cells, thus reinforcing the nexus between AD and T2DM and the contribution of insulin receptor “desensitization” with neuronal hypometabolism (215, 216).

Hypometabolism is a *bona fide* pre-symptomatic AD marker and is observed in scenarios including presenilin-1 mutation, apolipoprotein E4 subjects, and maternal AD background and in unrelated-to-age IR. As broadly described, central IR will certainly attain reduced brain glucose utilization, impaired cognitive performance, and white matter microstructural damages (217).

In other processes in which cognition declining is a sort of hallmark, like polycystic ovary syndrome, IR and white matter microstructural changes have also been identified (217). Furthermore, subjects undergoing prediabetes and diabetes exhibit cerebral hypometabolism and cognitive and visual memory impairments, which significantly correlate with smaller volumes of the brain (218). In line with this, hypometabolism appears doubly amplified in those cerebral AD-affected regions in individuals with T2DM as compared with non-diabetic controls (219). Taken together, all these pathologies are pathophysiologically bridged by a primary and major failure in glucose uptake and its posterior glycolytic transformation, which accounts for regional cerebral hypometabolism. Nonetheless, IR and hypometabolism are necessarily and significantly connected to mitochondrial dysfunction (203).

Accordingly, aerobic glycolysis appears to be an evolving biomarker of AD susceptibility, specifically for those brain regions in which Aβ deposition predisposes to a glucose metabolic rate beyond the physiological limits, which is irrespective to oxygen flow for ATP generation (220, 221). These studies inaugurated an era in which a myriad of researches judiciously focused on the role of glucose metabolism, mitochondrial enzymes, and OXPHOS.

AD transgenic mice and age-matched non-transgenic controls had evidenced decreased mitochondrial respiration, decreased pyruvate dehydrogenase (PDH) protein level, and activity along with increased oxidative stress as early as 3 months of age. Mitochondrial Aβ level was significantly elevated at 9 months. Embryonic neurons obtained from AD mice showed significantly decreased mitochondrial respiration and increased glycolysis, which mechanistically lines up with the hypometabolism that precedes AD diagnosis (222). Reports from the mid 1980–1990s addressed mitochondrial oxidative activity describing abnormal patterns of glucose metabolism and oxygen consumption in fibroblasts from AD patients. For instance, Curti et al. announced that these AD-derived fibroblasts show a far more acidic intracellular pH than the concurrent control, a reduction of complex IV cytochrome *c* oxidase activity, and a marked susceptibility to chemically induced oxygen consumption inhibition (223). Furthermore, hybrid cells (cybrid) demonstrated that AD platelet exhibits a deficit in cytochrome oxidase, which could be readily transferred to cells depleted of mitochondrial DNA (mtDNA). Ultimately, cybrid cell lines proved to recapitulate specific biochemical, molecular, and histologic AD traits, indicating that an mtDNA-related dysfunction might be pathogenically involved with AD (224). Interestingly, these biochemical changes were associated with abnormal mitochondrial morphology in cutaneous fibroblasts from LOAD patients, where they become significantly stretched and formed a highly meshed network (225). This is a meaningful message that again reinforces the notion that those AD-related mitochondrial changes are not a brain-limited episode but an alteration of energy metabolism homeostasis expressed in multiple tissues (226). A confirming finding was also made more than 20 years ago in PD patients' cutaneous fibroblasts. The mitochondrial bioenergetic changes observed in these PD skin cells and comparable with those from AD patients indicate that PD is not restricted to degenerating dopaminergic midbrain neurons but that, instead, these are hints of an early bioenergetic failure of extracerebral cells (227).

Other studies have described reduced activity of critical enzymes in energy metabolism, which may account for a primary ATP deficit [reviewed in (226)]. AD and other neurodegenerative conditions share a significant reduction of α-ketoglutarate dehydrogenase complex (228). These findings were further confirmed and extended when autopsy-collected brain samples displayed considerable reduction rates of PDH complex, isocitrate dehydrogenase, and the α-ketoglutarate dehydrogenase complex. On the contrary, the activity of succinate dehydrogenase (complex II) and malate dehydrogenase appeared increased. Importantly, these enzyme changes proved to have a tremendous clinical correlation, thus ratifying the impact of mitochondrial alterations for neuronal health (229). Similarly, using postmortem AD brain samples, Mauer et al. demonstrated the remarkable reduction of oxidative phosphorylation rates, particularly in cytochrome oxidase activity. This finding was highly appreciated as indicative of brain metabolic bioenergetics dyshomeostasis (230).

The results derived from the global microarray analysis conducted by Reddy et al., analyzing 11,283 cDNA clones from the cortex of APP transgenic mice at three age periods, concluded that genes related to mitochondrial energy metabolism and apoptosis appeared upregulated, as a compensatory response to the toxic effect induced by APP. These findings confirm that mitochondria and cellular energy metabolism are targeted by the harmful toxicity of Aβ (231). The picture portraying the failure in energy metabolism-associated enzymes was further validated in recent years, when it was shown that blood cells from AD patients exhibited lower expression levels of nuclear-encoded oxidative phosphorylation (OXPHOS) subunits and of those involved in the translation of mitochondrial-encoded OXPHOS (232). Furthermore, mitochondria isolated from AD triple transgenic mice models exhibit respiratory chain and phosphorylation system impairment, along with ultrastructural abnormal changes and oxidative imbalance, which accounted for a reduced ATP/ADP ratio (233). Other AD transgenic mice models are also identified by a cerebral impairment in ATP production related to mitochondrial dysfunction (234). Taken together, these evidences indicate that mitochondrial OXPHOS enzymes are largely compromised in AD accounting for a sort of Warburg-like effect, hypometabolism, and an energetic downfall.

The axonal transport system is responsible for moving mitochondria in both anterograde and retrograde directions and to the sites of synapses for an easy ATP provision. This is a critical event since mitochondria-derived ATP promotes axonal growth, calcium buffering, and importantly mitochondrial repair and recycling (235). Microtubules and motor proteins such as kinesin, dynein, and the OMM are involved in this axonal transport (201). Remarkably, these are ATP-dependent processes that demand a presumably optimal energy metabolism from glucose processing to mitochondrial-ATP output. A failure in this ATP-dependent generation process will impair axonal transport, which in turn drives the accumulation of toxic protein aggregates. Although the molecular operators behind mitochondrial transport inhibition in AD remains to be understood, a primary mitochondrial motility failure is tied to a deregulated fission/fusion rate, as to other relevant factors such as Aβ accumulation, hyper-phosphorylated Tau, and oxidative stress (236).

Mitochondria are dynamics organelles, with constant motility, and shape and size modifications within the cell. Accordingly, mitochondria exhibit an actual remodeling process influenced by environmental cues (237). Aging, neurodegenerative diseases, and stress situations are mitochondrial fragmentation-inducing factors (238).

As previously mentioned, Aβ fibrils and phosphorylated Tau tangles are “hallmarks of the disease” with a high nosogenic character and a shatterproof affinity for mitochondrial (239). Elegantly reviewed by Chakravorty and colleagues, the hierarchic position and the chronological window of Aβ and hyperphosphorylated Tau in relation to AD-mitochondrial damages are a field of intense and constant debate. There are evidences that place dysfunctional mitochondria in a pathology driving position, while other contradictory studies aim to Aβ and pathogenic Tau as the major forces toward mitochondrial dysfunction (240). Moreover, AD is a complex and multifactorial nature-progressive process in which numerous vicious circles and feedback loops are interconnected and cannot be discarded. It is known that among the injurious consequences of the neurotoxic Aβ and Tau duet, impairment of mitochondrial transport (241), abnormal mitochondrial motility (242), and intraneuronal maldistribution are described, especially associated with Tau overexpression (243). Furthermore, conclusions derived from triple AD transgenic mice compellingly emphasize the synergistic impact of Aβ and Tau in impairing mitochondrial OXPHOS, hindering ATP synthesis, over-generating ROS, reducing mitochondrial membrane potential, and altogether “mutilating” mitochondrial activity (244).

From the perspective of the classic “mitochondrial cascade hypothesis” (245) for the LOAD form, mitochondrial malfunction acts as a proximal event that causes Aβ deposition, synaptic degeneration, and intracellular NFT formation. Indeed, there are a wealth of studies showing that mitochondrial dysfunction is a frequent and decisive event in AD (246). The primary pathogenic role of mitochondria has been further nurtured in recent years. A crucial study indicated that despite that an individual's AD risk is determined by both parents, the maternal influence is far more profound than the paternal one (247). Similarly, another study found that platelet mitochondria cytochrome oxidase (COX) activity was lower in the children of AD mothers when compared with children of AD fathers (248). Ultimately, Aβ and pathogenic Tau may drive mitochondrial damage and dysfunction (240, 249).

Cellular metabolism is definitively impinged by mitochondrial remodeling including fission, fusion, and cristae modifications (238). Of note, imbalances between fusion, fission, and mitochondrial fragmentation are currently considered a major pathogenic driver and a distinguishable characteristic of AD and other neurodegenerative disorders (206, 238). These mitochondrial dynamics alterations in AD are driven by the activation of fission factors and downregulation of fusion factors to an impaired process of mitophagy (196). Mitochondrial fission is controlled by two GTPase genes: Fis1 and Drp1. Fis1 is essentially localized on the OMM and regulates mitochondrial fission. Most of Drp1 is localized in the cytoplasm, but a small part is found in the OMM especially upon activation where it oligomerizes and encircles the mitochondrion, thereby inducing fragmentation (250, 251). The pro-fission activity of Drp1 is regulated by phosphorylation, ubiquitination, SUMOylation and S-nitrosylation processes (252). Drp1 is also involved in apoptosis induction via BAX and cytochrome *c* release (238). Mitochondrial fragmentation is a hallmark of brain and fibroblasts derived from AD patients (206). This imbalanced pro-fission state in AD accounts for a reduction in mitochondrial metabolic surface, which leads to abnormal mitochondrial connectivity, impaired axonal transport, and importantly to a bioenergetic collapse. Under these circumstances, neuronal demise, synapse inhibition, and cognitive decline are the ultimate consequences (225, 253, 254).

Deeping on the abnormal mitochondrial dynamics in relation to Aβ oligomers in the AD brain samples, Manczak et al. succeeded in the seminal demonstration that Drp1 and Fis1 increase with AD pathology evolution and that monomeric and oligomerc Aβs interact with the pro-fission protein Drp1 in both human AD and AβPP/PS1 transgenic mouse-derived samples, which may ultimately translate in the AD characteristic exaggerated mitochondrial fragmentation and neuronal demise (237).

Mitochondria are gifted with a powerful quality control system that assists in mitochondrial and cellular homeostasis. This system, by mean of continuous surveillance, ensures an appropriate mitochondrial dynamics, adequate DNA synthesis, and normal protein folding. Furthermore, the system is committed to purge the population of wrecked mitochondria by a selective autophagy pathway, identified as mitophagy (250). This process of mitochondrial tagging for subsequent recycling seems to be activated upon the organelle's structural damaged and membrane potential fall down (240).

Recent evidences indicate that this process of damaged mitochondria purging is impaired in AD, which contributed to the intraneuronal accumulation of dysfunctional mitochondria (255, 256). *In vitro* models up to human samples document that a detrimental process of mitophagy is followed both by synaptic dysfunction and cognitive impairment by triggering Aβ and Tau accumulation, which are linked to oxidative damage and cellular energy deficits. An AD model developed in *Caenorhabditis elegans* proved that induction/resumption of mitophagy reversed the memory impairment. This study also demonstrated that mitophagy effectively reduced the levels of insoluble Aβ1–42 and Aβ1–40 and of extracellular Aβ plaques, via microglial phagocytosis and adjunctively by reducing inflammation (113). Thus, a reduction in the rates of mitophagy is an age hallmark and an AD badge, when it is associated with Aβ and hyperphosphorylated Tau (257).

Martin-Maestro et al. identified that LOAD patient-derived fibroblasts exhibited the same deficitary mitophagy, along with an abnormal accumulation of mitochondria, similar to that described in hippocampal neurons, reaffirming that mitophagy alterations are a sort of hallmark of sporadic AD (258).

**Figure 3:**
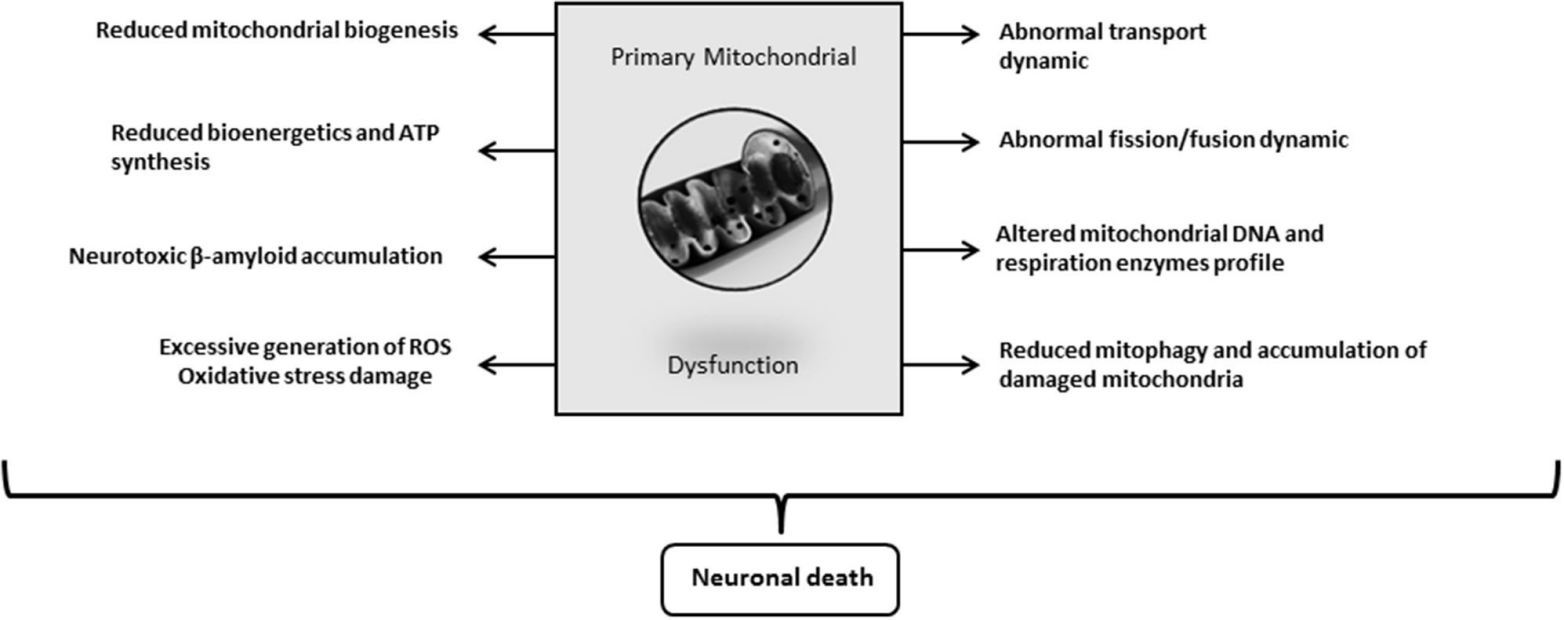
Alzheimer's disease (AD)-driven primary mitochondrial dysfunction. Although it is still debated “who drives who” along the course of molecular events leading to clinical AD, mitochondrial primary dysfunction is a well-founded hypothesis on the pathogenic cascade of AD. Mitochondrial dysfunction means that bioenergetic derangement is broad enough to trigger and run through a series of critical pathogenic ingredients of AD. A primary defect on mitochondrial physiology could arise from damaged mtDNA, deficit or failure of respiratory enzymes, alterations of oxygen uptake and handling, erroneous or insufficient tagging for a proper organelle purge, etc. The onset of an abnormal mitochondrial function irrevocably leads to neuronal death.

Again, the emblematic AD neurotoxins Aβ and Tau separately or synergistically attempt against mitochondrial function, including the processes of autophagy and mitophagy (241). Aβ progressively accumulates within mitochondria and impairs a variety of its functions (226, 259). Reddy's laboratory has extensively investigated the pathways underlying the pathogenic impact of Aβ and Tau against mitochondrial physiology in AD (257). This group demonstrated that Aβ and hyperphosphorylated Tau impair the normal autophagy and mitophagy processes by interacting with the mitochondrial fission protein Drp1 and eventually via the interaction of Aβ with PINK1/parkin (257). Collectively, mitochondrial functional collapse is a triggering and/or an exacerbation factor within the pathogenic chain encompassing IR, energy dysmetabolism, inflammation, oxidative stress, amyloid accumulation, and ultimately neuronal death (Figure 3). Pharmacological interventions addressed to preserve mitochondrial biogenesis and normal dynamics as to resume mitophagy may open encouraging therapeutic avenues for AD. (260).

The fact that free radicals may induce about 10,000 DNA alterations per cell per day (261) explains why oxidative stress is broadly recognized as a senescence and pathology driving factor (262). Furthermore, ROS/oxidative stress/mitochondrial dysfunction and inflammation *per se* and act as an indissoluble, interconnected nosogenic unit that links organismal aging, T2DM, obesity, and AD (263, 264).

Mitochondria, routinely identified as the “powerhouse of the cell,” are the major generator of members of the ROS family such as superoxide anion, hydroxyl and peroxyl radicals, and hydrogen peroxide (206, 265–267). Although the intracellular generation of ROS *per se* is an inevitable process, cells possess numerous defense systems to counter it. Furthermore, ROS are known to play numerous physiological roles under health conditions including the regulation of cell proliferation, growth factor receptor signaling, and intercellular communication (268). ROS overproduction under certain pre-morbid or morbid scenarios acts as a silent destructive influence that overcomes cell detoxification mechanisms and engenders and/or amplifies multiple molecular damage nodes. As described by Reddy's group, this pro-oxidative imbalanced milieu is associated with damages of mitochondrial and cellular lipids, DNA, and proteins (269).

Of note, mitochondrial dysfunction/oxidative stress is considered one of the alternative mechanisms nurturing hyperinsulinemia/IR. It is conceived that an unbalanced generation of mitochondrial ROS as a primary event directly hinders insulin signal transduction by IRS inhibitory phosphorylation via JNK activation. ROS alternatively may synergistically trigger intracellular inflammatory signaling pathways (via NF-kB) that ultimately impair insulin receptor system activation, accounting for a persistent inflammation-mediated IR (270–272).

ROS are capable of destroying the inner mitochondrial membrane system and consequently the ETC, which generates an additional destructive vicious circle given by further ROS overproduction, extensive oxidative stress damages, and major ETC failure. Under this scenario, ATP synthesis is impaired, and mitochondria become functionally collapsed and a self-destructive organelle (273).

Compelling evidences attests the broad horizon of pathogenic commitment of mitochondrial dysfunction/oxidative stress in AD development and progression. Similarly, numerous studies document how Aβ pathogenically cooperates with ROS, enters into the mitochondria, dismantles the ETC system aggravating the ROS leakage, damages the mitochondrial cisternae system, and eventually extinguishes ATP cellular production. The richness of data supporting these exciting findings has incited the search for mitochondria-targeted antioxidants [for review, see (274)]. The cellular damage brought by oxidative stress in AD is far-reaching, expansive, perpetuative, and long-lasting. Consequently, we have approached to summarize oxidative stress consequences, as follows: (1) impairs mitochondrial dynamics and increases the rate of mitochondrial fission, which further amplifies the bioenergetic mitochondrial dysfunction and neuronal demise (275); (2) impairs the activity level of intermediary metabolism enzymes as aconitase, glutamine synthetase, creatine kinase, PDH, and α-ketoglutarate dehydrogenase as described for AD brains and cells exposed to Aβ (276–278), leading to further bioenergetic dyshomeostasis (279); (3) induces cerebral and peripheral inflammation by stimulating the release of mitochondrial components into the cytosol, which activates innate immune response mechanisms, via an inflammasome-dependent pathways (238); (4) the recognition of these mitochondrial products as DAMPs induces an interferon-like response that amplifies and polarizes inflammation, thereby altering mitochondrial dynamic, membrane permeability, and the bioenergetic work (238); (5) ROS and lipid peroxidation contribute to phosphorylate Tau with the ensued enhancement of more SOD deficiency and exacerbation of the mitochondrial dysfunction (269); (6) given that mtDNA is relatively unprotected, its proximity to the main source of ROS and the unavailability of an efficient repair system turn mtDNA far more vulnerable to chemical damages than its nuclear counterpart. MtDNA oxidative damage is associated with a variety of mutations that mostly affect the core enzymes of the bioenergetic activity. Therefore, the consequences are predictably disastrous (196). (7) The ROS-induced failure in mitochondrial membranes stability potential predisposes to the opening of the mitochondrial permeability transition pore; eventually, this allows for cytochrome *c* and other pro-apoptotic proteins to leak, triggering the apoptotic caspase cascade (280); (8) ROS also exhibit a synergistic pathogenic cooperativity with Aβ, which further intensifies mitochondrial and neuronal damages and neurotoxicity (249, 273).

Taken together, these evidences provide a substantial foundation to consider mitochondrial functional impairment and ROS/oxidative stress as a critical node in AD pathology given its commitment in neuronal energetic bankruptcy. It is far more than convincing that ROS/oxidative stress contributes to and derives from IR and that it plays a critical role in neuronal damage expansion and cognitive deficit amplification (281). Finally, mitochondrial transplantation has recently emerged as a hopeful therapeutic tool in order to restore neuronal homeostasis, survival, connectivity, and regeneration in neurodegenerative diseases. It still remains challenging how and when to precisely deliver the salutary mitochondrial message to the diseased substrate (282).

## Concluding Remarks

Interestingly, numerous phenotypic traits support the notion that AD is not a CNS-circumscribed disease. Similarly, this seems to be the truth for other devastating neurodegenerative process. The pathogenesis of AD is convoluted and multifactorial, which hampered the identification of critical molecular therapeutic targets for a definitive mitigation. A simple journey across the years of research and publications denotes that although there have been undeniable progresses in understanding the original molecular mechanisms underlying this complex disease, dozens of gaps still remain to be filled, not to mention the imperative of identifying the hierarchic pathophysiological sequence of events. Understanding this hierarchical order and its related molecular signaling pathways will advance therapeutic approaches. Conceivably, causes and consequences will be judiciously segregated and grouped. Contemporary research in AD is multifactorial and productive; and its findings and output are likely comparable with those seen in cancer research.

**Figure 4:**
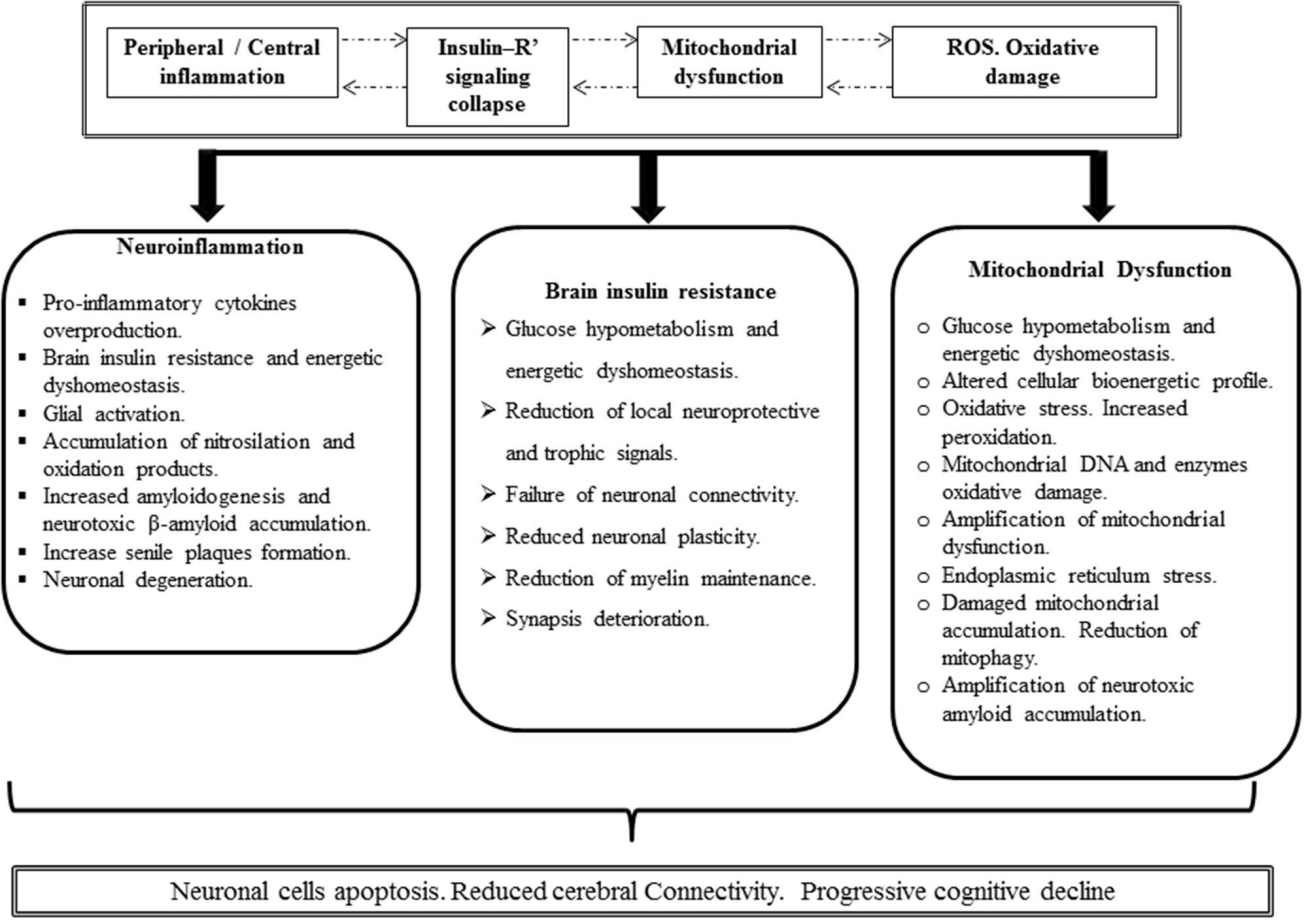
Putative Alzheimer's disease (AD) pathogenic integrative mechanism. Inflammation (either peripheral or central), IR, mitochondrial functional impairment, and the excessive production of ROS with the ensued oxidative damage are concatenated and mutually cooperate as pathogenic drivers. It is likely that one of these four ingredients can consistently drive to others so that a hierarchy of events may turn irrelevant. Inflammation breaches the BBB with the downstream consequences, elicits a neurotoxic environment, and triggers glial and astrocyte activation with further cytokine spillover. Oxidative stress may be an inflammation by-product that in turn amplifies inflammation. Concurrently, free radicals may turn on inflammatory pathways. Moreover, proinflammatory cytokines trigger IR by imposing a loss-of-function pattern of phosphorylation in insulin receptor substrate 1. Importantly, obstruction of cerebral insulin physiology translates neuronal cell vulnerability to inflammation, mitochondrial impairment, and oxidative stress and may be an amplifying factor to each of these nosogenic links. Furthermore, the functional collapse of cerebral insulin leads to increase of β amyloid accumulation, neurotoxicity, and ultimately neuronal demise. Impaired mitochondrial function either by an acquired or inherited defect or via β amyloid accumulation may turn sufficient to dismantle cerebral cell homeostasis.

There is an emerging constellation of novel targets and potential therapeutic candidates that may revolutionize future medical approaches, ranging from intracerebral iron accumulation to an anticipated epigenetic dysregulation of a variety of genes in asymptomatic patients. The presence of chronic inflammation, IR, oxidative stress, and brain/peripheral mitochondrial dysfunctions are, moreover, major and repeatedly identified drivers of AD pathology. Whether primary or secondary in origin, brain chronic IR may underlie the reputed cerebral hypometabolism and consequently may initiate a neurodegenerative cascade that translates in mitochondrial failure and neuronal energetic dysfunction with the adjacent oxidative stress and neuroinflammation. The pathogenic responsibility of this cascade is well-sustained, and glucose hypometabolism correlates with symptoms of severity, synaptic dysfunction, and cognitive impairment. Impaired cerebral glucose metabolism is also invariantly reported in type 2 diabetes, and its consequences can readily account for most of the structural and functional anomalies of AD. A deficient insulin and IGF-I activity may link the two diseases given the dramatic relevance of the hormone in superior nerve structures as in βA and Tau metabolism and turnover. We consequently consider the quartet of chronic inflammation, IR, oxidative stress, and mitochondrial dysfunction in a proximal position to neurotoxic amyloid deposits. These aspects are depicted in Figure 4 in an integrative fashion.

Irrespective to the number of questions that still remain unanswered as how to prevent proinflammatory cytokine endovascular elevation or how the peripheral-derived proinflammatory molecules impact on brain cells, the truth is that for both AD and T2DM, IR is greatly impinged by IRS-1 functional obstruction. Solid science from more than 20 years ago attests that proinflammatory cytokines induce central and peripheral IR via increased IRS-1 serine phosphorylation with a concomitant decrease of activating phosphorylation on tyrosine residues. In close resemblance to T2DM, AD-derived exosome biomarkers reflect the inflammation-associated imprinting on insulin receptor signaling components. Thus, the similitude of these basic and primary underlying mechanisms presupposes the possibility of a common “druggable” target. Druggable goals may entail how to prevent inflammation and/or how to elude the consequences of inflammation over the insulin receptor signaling system. New therapeutic perspective can also target the AD-diseased mitochondria. Mitochondrial dysfunction may precede the onset of AD. Again, to what extent IR leads to mitochondrial dysfunction remains elusive. Moreover, AD as a pathological and overexpressed form on an organ-specific aging may also incite to target PGC-1α in its intersection with DNA damage, mitochondrial turnover, and functional homeostasis. These are all perhaps future druggable targets. Furthermore, finding the pharmacological tools to ensure appropriate diseased mitochondrial purge could theoretically ensure neuroprotection and age-associated cognitive decline. Accordingly, a sort of “integral mitochondriotherapy” tool is called to show up in the avenues of brain bioenergetics correction. Harnessing brain energy metabolism will provide novel and effective preventive and therapeutic alternatives for AD and other neurological and psychiatric disorders.

## Author Contributions

JB-A, GG-N, MV-S, and PV-S contributed to the project conception. JB-A designed the study and wrote the first draft of the manuscript. DG, NR-R, AG-O, and JB-S contributed to the analysis and interpretation of data. MB-V helped during the preparation of the manuscript and the whole editorial process. JB-A and JB-S contributed equally to data collection. All authors contributed to interpretation, critically reviewed the manuscript, approved the definitive version of the manuscript, and agreed to be accountable for all aspects of this paper.

## Conflict of Interest

The authors declare that the research was conducted in the absence of any commercial or financial relationships that could be construed as a potential conflict of interest.

## Abbreviations

AD: Alzheimer's disease
APP: amyloid precursor protein
APP/PS1: amyloid precursor protein/presenilin-1
βA: beta-amyloid
βAOs: beta-amyloid oligomers
BBB: blood–brain barrier
CNS: central nervous system
COX: cytochrome oxidase
CSF: cerebrospinal fluid
DAMP: damage-associated molecular pattern
Drp-1: Dynamin-related protein 1
ER: endoplasmic reticulum
ETC: electron transport chain
FAD: familial AD
FDG-PET: fluorodeoxyglucose positron emission tomography
GLUT-3: glucose transporter 3
GLUT-4: glucose transporter 4
GSK-3β: glycogen synthase kinase-3β
INF-γ: interferon gamma
IGF-I: insulin-like growth factor type I
IGF-II: insulin-like growth factor type II
IL-1: interleukin-1
IL-6: interleukin-6
IR: insulin resistance
IRS: insulin response substrate
JAK-STAT: Janus kinase signal transduction and activator of transcription
JNK: c-Jun N-terminal kinase
LOAD: late onset AD
MAPK: mitogen-activated protein kinase
M-CSF: macrophage colony-stimulating factor
mtDNA: mitochondrial DNA
NFT: neurofibrillary tangle
NLRP3, NOD-, LRR-: and pyrin domain-containing protein 3
OMM: outer mitochondrial membrane
PD: Parkinson's disease
PDH: pyruvate dehydrogenase
PDHC: pyruvate dehydrogenase complex
PGC-1α: peroxisome proliferator-activated receptor gamma coactivator-1 alpha
PI3K: phosphatidylinositol 3-kinase
PPAR-γ: peroxisome proliferator-activated receptor gamma
pTau: phosphorylated Tau
RAGE: receptor for advanced glycation end products
ROS: reactive oxygen species
SAD: sporadic AD
STZ: streptozotocin
TGF-β: transforming growth factor beta
TLR: toll-like receptors
TNF-α: tumor necrosis factor alpha
T2DM: type 2 diabetes mellitus.
